# Comparison of risk factors for seropositivity to feline immunodeficiency virus and feline leukemia virus among cats: a case-case study

**DOI:** 10.1186/s12917-015-0339-3

**Published:** 2015-02-10

**Authors:** Bimal K Chhetri, Olaf Berke, David L Pearl, Dorothee Bienzle

**Affiliations:** Department of Population Medicine, Ontario Veterinary College, University of Guelph, Guelph, ON Canada; Department of Mathematics and Statistics, University of Guelph, Guelph, ON Canada; Institute of Biometry, Epidemiology and Information Processing, University of Veterinary Medicine Hanover (Foundation), Hanover, Germany; Department of Pathobiology, Ontario Veterinary College, University of Guelph, Guelph, ON Canada

**Keywords:** Cat, Epidemiology, Retrovirus, FIV, FeLV

## Abstract

**Background:**

Feline immunodeficiency virus (FIV) and feline leukemia virus (FeLV) are reported to have similar risk factors and similar recommendations apply to manage infected cats. However, some contrasting evidence exists in the literature with regard to commonly reported risk factors. In this study, we investigated whether the known risk factors for FIV and FeLV infections have a stronger effect for either infection. This retrospective study included samples from 696 cats seropositive for FIV and 593 cats seropositive for FeLV from the United States and Canada. Data were collected during two cross sectional studies, where cats were tested using IDEXX FIV/FeLV ELISA kits. To compare the effect of known risk factors for FIV infection compared to FeLV, using a case-case study design, random intercept logistic regression models were fit including cats’ age, sex, neuter status, outdoor exposure, health status and type of testing facility as independent variables. A random intercept for testing facility was included to account for clustering expected in testing practices at the individual clinics and shelters.

**Results:**

In the multivariable random intercept model, the odds of FIV compared to FeLV positive ELISA results were greater for adults (OR = 2.09, CI: 1.50-2.92), intact males (OR = 3.14, CI: 1.85-3.76), neutered males (OR = 2.68, CI: 1.44- 3.14), cats with outdoor access (OR = 2.58, CI: 1.85-3.76) and lower for cats with clinical illness (OR = 0.60, 95% CI: 0.52-0.90). The variance components obtained from the model indicated clustering at the testing facility level.

**Conclusions:**

Risk factors that have a greater effect on FIV seropositivity include adulthood, being male (neutered or not) and having access to outdoors, while clinical illness was a stronger predictor for FeLV seropositivity. Further studies are warranted to assess the implications of these results for the management and control of these infections.

## Background

Infections with feline immunodeficiency virus (FIV) and feline leukemia virus (FeLV) are two of the most common and important infectious diseases of cats [1,2]. The most common mode of transmission of FIV is through bites [3,4]. FeLV infection is also commonly acquired via the oro-nasal route through mutual grooming, nursing or sharing of dishes apart from bites [3]. The known risk factors for acquiring these infections are male sex, adulthood and exposure to outdoors, whereas being neutered and indoor lifestyle are known protective factors [5]. However, the relative importance attributed to age, outdoor exposure and sex among infected cats is variable in the literature. Some studies indicate that FeLV infections are age-dependent [6] and primarily acquired by “friendly” cats through prolonged close contact between virus shedders and susceptible cats through mutual grooming, sharing of food and water dishes, and use of common litter areas [3]. However, other studies have indicated adulthood [1,7], outdoor lifestyle [1,7], being not neutered [8], and fighting [8,9], factors commonly associated with FIV, to also be associated with FeLV infection. Thus, further research is necessary to investigate the relative importance of these factors to help in management and prevention of these infections.

Case–control studies are often used in analytical epidemiology to examine the strength, magnitude and direction of associations between exposure variables and an outcome of interest [10]. Case-case studies are a variant of case–control studies when the disease of interest can be sub-classified in two or several groups that may have distinct risk factors [11]. A case–case study differs from a case–control study in that the comparison group (or controls) are also selected among the cases, typically with same disease but a different strain or serotype, from the same surveillance system [11]. The case-case study approach has been used increasingly in epidemiology e.g. to compare risk factors for two subtypes of the same disease with the goal of ascertaining relative importance of risk factors for either subtype [11]. The main advantage of the case-case design is its ability to limit selection and information biases: control cases have similar clinical features, are identified through the same system and are subject to the same biases as cases [11,12]. The goal of this study was to assess the relative importance of known risk factors between the two common feline retroviral infections, FIV and FeLV, using the case-case study approach.

## Methods

### Data source and study participants

A dataset consisting of diagnostic test results from 29,182 cats tested for FIV and FeLV between August and November of the year 2004 and 2007 from the United States (US) and Canada was obtained from two previous cross-sectional studies [1,7]. The cats included in these studies were conveniently sampled from veterinary clinics and animal shelters across 40 contiguous states of the US and 9 Canadian provinces encompassing 641 US zip codes and Canadian forward sortation areas in 346 US counties and Canadian Census Divisions. The first study investigated cats in the US and Canada while the second study was restricted to the Canadian cat population.

Data collection has been described elsewhere [1,7]. Briefly, potential veterinary clinic participants in the US were identified from the membership roster of the American Association of Feline Practitioners (AAFP) as well as from the list of all individuals who had purchased test kits for FIV and FeLV. Potential animal shelter participants (including cat rescue organisations, and groups participating in Trap-Neuter-Release (TNR) programs) were derived from various Internet directories [1]. In Canada, potential veterinary clinic and animal shelter (including cat rescue programs and feral cat programs in Canada) participants were identified as all those who had purchased test kits for FIV or FeLV or submitted samples to a diagnostic laboratory [7]. Potential study participants were sent an invitation letter to participate in the study. Enrolled participants submitted the diagnostic results for FIV and FeLV along with information on age, sex, neuter status, outdoor exposure, health status and test date using a standard reporting form. The testing and reporting was performed from August to November 2004 for the American and Canadian participants in the first study and from August to November 2007 for the Canadian participants in the second study.

### Testing protocol

The testing for FIV and FeLV was carried out in-house or in-laboratory employing a commercially available ELISA (SNAP® Combo FeLV antigen/FIV antibody, PetCheck® FIV Antibody and PetCheck® FeLV Antigen; IDEXX Laboratories) using whole blood, serum or plasma. The manufacturer reported sensitivity and specificity of the assay for detecting FeLV antigen of 97.6% and 99.1%, and for detecting FIV antibodies of 100% and 99.5%, respectively. Confirmatory testing was not performed.

### Covariate information

Information on postal code of testing facility, type of testing facility (clinic or shelter), age of the cat (juvenile [<6 months] or adult), sex and neuter status (sexually intact female, spayed female, sexually intact male or neutered male), access to outdoors (indoors or outdoors) and general health at time of testing (healthy or sick) was also retrieved from the dataset (Table 1).

**Table 1:** **Descriptive characteristics of the FIV and FeLV seropositive cat populations**

**Factors**	**Total samples**	**FeLV+**	**FIV+**
		**n (%, 95% CI)**	**n (%, 95% CI)**
**Testing Site**			
Veterinary Clinic	1064	503 (47.3, 44.2-50.3)	561 (52.7, 49.7-55.8)
Shelter	225	90 (40.0, 33.5-46.7)	135 (60.0, 53.3-66.5)
**Age**			
Juvenile	281	165 (58.7, 52.7-64.5)	116 (41.3, 35.5-47.3)
Adult	1008	428 (42.5, 39.4-45.6)	580 (57.5, 54.4 -60.6)
**Sex**			
Male Intact	469	174 (37.1, 32.7-41.6)	295 (62.9, 58.4-67.3)
Male Neutered	380	147 (38.7, 33.8-43.8)	233 (61.3, 56.2-66.2)
Female Intact	262	167 (63.7, 57.6-69.6)	95 (36.3, 30.4-42.4)
Female Spayed	178	105 (59.0, 51.4-66.3)	73 (41.0, 33.7-48.6)
**Outdoor Exposure**			
No	217	126 (58.1, 51.2-64.7)	91 (41.9, 35.3-48.8)
Yes	1072	467 (43.6, 40.6-46.6)	605 (56.4, 53.4-59.4)
**Health Status**			
Healthy	708	303 (42.8, 39.1-46.5)	405 (57.2, 53.5-60.9)
Ill	581	290 (49.9, 45.8-54.1)	291 (50.1, 45.9-54.2)

### Selection of study subjects: FIV and FeLV case groups

Cats testing positive for FIV antibodies in ELISA were compared to cats testing positive for FeLV antigen with all the cats having been tested for both infections. Cats were excluded from further analysis in this study if they tested positive for both FIV and FeLV.

### Logistic regression

Logistic regression models were fit to model the logit of the probability of FIV seropositivity as a function of predictor variables age, sex/neuter status, outdoor exposure, health status and testing facility in a random intercept logistic regression model framework.

#### Univariable analysis

Variables were screened for inclusion into the multivariable logistic regression model by fitting univariable logistic regression models, without random intercepts, and those predictor variables with a liberal significance level (α = 0.2) were selected. However, care was taken not to remove predictor variables that were deemed clinically relevant. Since all the predictor variables were categorical (i.e. indicator variables), the significance in the model of each group of the predictors was analyzed by applying a likelihood ratio test. Collinearity among the predictor variables with significant unconditional association with FIV seropositivity was assessed by using the Spearman rank-correlation test. When two variables were collinear, the one with the smaller P-value was considered for further multivariable analysis while the other was removed.

#### Multivariable analysis

Backward selection was employed for multivariable model building and covariate removal from the model was based on the following criteria: (1) the highest non-significant P-value (with significance level α = 0.05); (2) a likelihood ratio test of the model with and without the variable that was non-significant and (3) the variable was not an important confounder for other variables in the model. A confounder was a non-intervening covariate whose removal from the model resulted in greater than 20% change in coefficients on the log-odds scale for any of the remaining variables in the model. Two-way interaction terms among type of testing facility, health status, outdoor exposure, age and sex were also assessed for statistical significance. However, interaction terms were dropped when these led to sparse cells and unrealistic estimates. Multicollinearity was tested among screened variables in the multivariable logistic regression model by estimating the variance inflation factor (VIF). All variables with a VIF value of 10 or above were considered to indicate multicollinearity, assuming that this was not due to variable construction (e.g. interaction terms) [10]. Non-nested multivariable models were compared using the Akaike Information Criterion (AIC), and the model with lowest AIC value was considered to be better fitting.

To account for clustering by testing facility (i.e. clinics or shelters), all multivariable logistic regression models included a random intercept for testing facility. Relevance of the random effect term for facility ID was assessed by inspection of the variance component. A simpler model (without random effects) was chosen when the variance component was close to zero [13]. Fit of the random effect model was assessed visually by plotting the QQ-plots of the Best Linear Unbiased Predictors (BLUPs) against the normal scores [10].

The random intercept models were fit in statistical software R (lme4 package) and Stata (xtmelogit) by seven point Gauss-Hermite adaptive quadrature method [14,15], using complete cases (i.e., any observations with missing values excluded from the analysis). However, the point estimates from the final model were compared to the same model fit with missing values (coded as unknown) to observe any gross deviation in direction and magnitude.

The Research Ethics Board at the University of Guelph did not require ethics approval for this study because secondary data was used without either patient or owner identifiers.

## Results

### Descriptive statistics

Table 1 presents the descriptive statistics of FIV and FeLV cases cross-tabulated by risk factors. The total number of cases included in this study was 1289. Out of these retroviral cases, 696 tested positive for FIV and 593 for FeLV.

### Logistic regression analysis

All covariates met the inclusion criteria for multivariable modeling as explained above (Table 2). The final multivariable random intercept logistic regression model included the covariates/predictors age, sex/neuter status, outdoor exposure, and health status of cats (Table 3). The odds ratio (OR) associated with each variable is adjusted for the remaining variables in the model. No significant interactions were detected between the variables that remained in the final multivariable model.

**Table 2:** **Results of univariable logistic regression analysis of risk factors for infection to FIV compared to FeLV**

**Variable**	**β**	**OR (95% CI)** ^**a**^	**P (Wald test)**	**P (LR test)** ^**b**^
**Type**				0.046
Clinic		Ref.		
Shelter	0.296	1.34 (1.00,1.80)	0.047	
**Age**				<0.001
Juvenile		Ref.		
Adult	0.656	1.93 (1.47,2.52)	<0.001	
**Sex and neuter status**				<0.001
Intact Female		Ref.		
Spayed Female	0.201	1.22 (0.83,1.81)	0.314	
Intact Male	1.092	2.98 (2.18,4.08)	<0.001	
Neutered male	1.025	2.79 (2.01,3.86)	<0.001	
**Outdoor Exposure**				<0.001
Indoor		Ref.		
Outdoor	0.584	1.79 (1.33,2.41)	<0.001	
**Health Status**				0.006
Healthy		Ref.		
Ill	−0.287	0.75 (0.60,0.94)	0.011	

^a^: Odds Ratios and 95% Confidence Intervals.

^b^: Likelihood Ratio Test p-value.

**Table 3:** **Results of the final mixed effects multivariable logistic regression model for analysis of risk factors for infection with FIV compared to FeLV**

**Variable**		**OR (95% CI)** ^**a**^	**P (Wald test)**
**Age**			
	Juvenile	Ref.	
	Adult	2.09 (1.50-2.92)	<0.001
**Sex and neuter status**			
	Intact Female	Ref.	
	Spayed Female	1.35 (0.66-1.65)	0.227
	Intact Male	3.14 (1.85-3.76)	<0.001
	Neutered Male	2.68 (1.44-3.14)	<0.001
**Outdoor Exposure**			
	Indoor	Ref.	
	Outdoor	2.58 (1.74-3.93)	<0.001
**Health Status**			
	Healthy	Ref.	
	Ill	0.60 (0.52-0.90)	<0.001
**Random effects**	Variance	SE	95% CI
At testing facility level	1.196	0.25	1.06-1.77

^a^: Odds Ratios and 95% Confidence Intervals.

The odds of cats being seropositive for FIV relative to FeLV was significantly greater for adult cats than juvenile cats (Table 3). Similarly, the intact and neutered males were significantly more likely to be seropositive for FIV than FeLV compared to intact females. The odds of being seropositive for FIV relative to FeLV was not significantly different between intact and spayed females based on the Wald test. Compared to cats kept indoors, cats with known outdoor exposure had higher odds of being seropositive for FIV relative to FeLV. For clinically ill cats, the odds of being seropositive for FIV relative to FeLV were smaller compared to cats without clinical illness.

The variance components obtained from the multilevel logistic regression model for the individual level and clinic/shelter level were 3.29 and 1.16, respectively.

A random effects logistic regression model was deemed appropriate due to clustering expected for cats tested within the same facility and because the variance of the random effect was 3.29, which given the associated small standard error was interpreted as the variance being different from zero (Table 3). Normal quantile plot of the BLUPs indicated no gross deviation from normality.

## Discussion

This case-case study is based on cross-sectional or prevalence data and thus generally not suited to identify risk factors. However, only known risk factors [3] were evaluated in this study with respect to their importance as risk factors for infection with FIV compared to FeLV. The results from this study imply that risk factors commonly associated with FIV and FeLV differ in their relative effects for these two diseases. For example adult, male, or outdoor cats are more likely to be seropositive for FIV than FeLV when compared to juvenile, female or cats kept exclusively indoors. In contrast, neuter status was not significantly different for either infection. Further, whether cats were tested at clinics or shelters was not different for these infections.

Most FIV infections are acquired as a consequence of bite wounds inflicted by an infected cat, presumably through inoculation of virus or virus infected cells [16,17]. Although, vertical transmission of infection from queen to kitten may occur, it is considered rare [18]. Adult, male, outdoor exposed cats would be expected to have a higher likelihood of getting infected with FIV due to higher likelihood of encountering infected cats, and being prone to aggression and territorial fights. On the contrary, most FeLV infections occur after oro-nasal spread of the virus from the viremic cats [17,19-22]. FeLV infection, thus, is a concern in cats that are “friendly” and in close contact with infected cats through nursing, mutual grooming or sharing dishes, but also through bites [3].

This study found a higher likelihood of FIV (compared to FeLV) seropositivity in adults. In contrast to FIV, FeLV is reported to be age dependent with older cats becoming increasingly resistant to infection [23,24]. Of note, however, is the fact that while age at acquisition is similar for both infections, FeLV can cause serious, often fatal, disease. As a result, FeLV-infected cats have shorter survival rates [25,26] and not many live to adulthood, while most FIV infected cats do.

Higher probability of infection can be expected in males compared to females for FIV [9,27-37]. But for FeLV, most studies did not find an association between sex and seropositivity [28,38] except for a single report [9]. The association between male sex and FIV infection has been primarily related to increased risk of infection transmission due to greater predisposition of males to exhibit territorial behaviour involving fighting. In this study, regression models included contrasts to compare the likelihood of seropositivity of FIV between intact and neutered male cats as well as between intact and spayed female cats. Although, compared to females, males were found to be more likely to test seropositive for FIV compared to FeLV, no significant differences were evident between intact and neutered cats for the same sex (Table 3 and 4). Various studies have reported an association between neutering and lower risk of infection of FIV and FeLV among domestic cats [8]. However, there are reports suggesting that neutering and spaying have no significant effect on the prevalence of FIV [27,39,40] and that such cats still retain territorial aggressiveness [39,40]. It should be noted that when a predictor is common to both FIV and FeLV, due to its inherent design, a case–case study might not detect a difference between the two case groups. In other words, if neutering were significantly associated with both FIV and FeLV seropositivity, this study design would not detect it. Since a higher likelihood of seropositivity was found in intact compared to neutered cats when non-infected cats were included [1,7], it is possible that sterilization characteristics are not different between FIV and FeLV infected cats.

**Table 4:** **Contrasts for the association between FIV seropositivity and sex/neuter characteristics compared to FeLV seropositivity**

**Contrast**	**OR (95% CI)** ^**a**^	**P (Wald test)**
Spayed female vs. Intact male (Referent category)	0.43	<0.001
Neutered male vs. Intact male (Referent category)	0.85	0.374
Neutered male vs. Spayed female (Referent category)	1.98	<0.005

^a^ Odds ratio for the contrast after adjusting for age, outdoor exposure and health status.

Cats were more likely seropositive for FIV than FeLV when exposed to outdoors than being indoors. This finding suggests that outdoor exposure is more important to acquire FIV infection than FeLV. Considering prevalence studies where non-infected cats were included, there seems to be consensus that the probability of FIV infection is higher for cats that roam outdoors [9,41] due increased opportunity for transmission via fights. In contrast, the relationship between outdoor exposure and FeLV infection is not very clear.

Healthy cats were more likely to test positive for FIV than FeLV compared to cats presenting as ill at the time of testing. Both viruses induce immunodeficiency, but FeLV is more rapidly pathogenic and its effects manifest sooner and include other disease conditions [26]. FIV infection causes gradually developing immunodeficiency and has only a minor impact on lifespan. Therefore, cats with FeLV are more likely to be presented with illness. This contributes to more sick cats testing FeLV positive rather than FIV positive.

The variance components of the random effects model indicate that some degree of clustering was evident at testing facility (ICC = 0.26) suggesting that FIV seropositive status compared to FeLV was not independent of shelter or clinic.

A few important limitations of the case-case study design in the context of this study merits attention. For a detailed account of pros and cons of case-case studies in general the reader is referred to McCarthy and Giesecke [11]. This study entailed comparison of FIV seropositive cats to FeLV seropositive cats with regard to known risk factors and explored the strength of their effects between the two infections. Therefore, care should be taken before extrapolating results of this study to the general population with non-infected cats. The risk factors that are common to both comparison groups tend to be underestimated or unidentified in a case-case study [11,12]. Since the study does not include a disease-free population, the odds ratios can only be interpreted as the odds of exposure to one disease group (FIV) in reference to the other (FeLV), and do not provide the estimate of the association between a risk factor and disease in the general population [42,43].

## Conclusion

In conclusion, while similar risk factors have been reported for both FIV and FeLV infection, this study demonstrated, through comparison of one infection with the other, that adulthood, being male (neutered or not) and having access to outdoors are of greater importance to FIV seropositivity compared to FeLV. Clinical illness was a stronger predictor for FeLV seropositivity. Further studies are warranted to assess the implications of these findings in regard to the management and control of these infections.

## Abbreviations

FIV: Feline Immunodeficiency Virus
FeLV: Feline Leukemia Virus
US: United States
ELISA: Enzyme Linked Immunosorbent Assay
AAFP: American Association of Feline Practitioners
TNR: Trap-Neuter-Release
VIF: Variance Inflation Factor
AIC: Akaike Information Criterion
BLUPS: Best Linear Unbiased Predictors
